# Artificial Intelligence Assistance Does Not Provide Immediate Benefit for Right Upper Quadrant Window Acquisition

**DOI:** 10.7759/cureus.106235

**Published:** 2026-03-31

**Authors:** Jacob Lenning, Corey L Garrison, Aaron R Mahoney, Paul F Thanel

**Affiliations:** 1 Emergency Medicine, Western Michigan University Homer Stryker M.D. School of Medicine, Kalamazoo, USA

**Keywords:** artificial intelligence and education, focused assessment with sonography for trauma (fast), medical education and training, point-of-care-ultrasound, ultrasound education

## Abstract

Introduction: Many point-of-care ultrasound (POCUS) machines now incorporate artificial intelligence (AI)-assisted acquisition features; however, their usefulness for the Focused Assessment with Sonography for Trauma (FAST) exam remains poorly defined. This repeated-measures study aimed to determine the immediate combined effect of AI auto-labeling and auto-grading on acquisition time and image quality of the right upper quadrant (RUQ) FAST exam window, as obtained by both novice and experienced emergency medicine (EM) physician trainees.

Methods: Fourteen novices with limited POCUS training during medical school and 10 second- and third-year EM residents recorded RUQ windows with and without AI assistance on three standardized patients in randomized order. Acquisition time (in seconds) was compared using the Mann-Whitney U test. A chi-square analysis was used to compare the proportion of recordings meeting each of the three image quality criteria: visibility of all essential structures, correct image plane, and proper probe orientation.

Results: The median (interquartile range) acquisition time was significantly longer with AI assistance for both novice trainees (85 (91) vs 53 (59) seconds; p < 0.01) and experienced trainees (44 (29) vs 28 (21) seconds; p < 0.01). All RUQ image quality criteria were significantly more likely to be met by experienced trainees than by novices. No significant differences in image quality were observed with versus without AI assistance within either group.

Conclusion: The immediate effect of AI auto-labeling and auto-grading was an increase in acquisition time among both novice and experienced physician trainees obtaining RUQ FAST exam windows. AI assistance was not associated with an immediate improvement in image quality among either group. These findings suggest that such AI features are unlikely to aid physician trainees in acquiring RUQ FAST exam windows during isolated attempts.

## Introduction

The application of artificial intelligence (AI) has led to the latest advancements in many areas of healthcare, including point-of-care ultrasound (POCUS). Unique from other imaging modalities, POCUS is rather operator-dependent because it requires bedside image acquisition in addition to image interpretation by the same user [1]. Therefore, many POCUS machines now incorporate AI features to assist users with both the acquisition and interpretation components of POCUS [2]. Image acquisition is supported with real-time probe guidance, real-time image grading, automatic labeling, and automatic saving. Interpretation is aided by automatic pathology detection and automatic measurements. Due to the operator dependency specific to POCUS image acquisition, the helpfulness of AI acquisition features has become an important focus of the latest POCUS medical education research [3].

Ample evidence supports the use of POCUS machines incorporating AI auto-grading, auto-labeling, and probe-guidance features to assist users with obtaining cardiac POCUS windows. Multiple studies demonstrated that novice users with AI assistance can achieve diagnostic-quality cardiac POCUS windows [4-6]. One study even reported that image quality by novice users with AI assistance was comparable to image quality by expert sonographers [7]. Other studies demonstrated better image quality among novice users with AI assistance compared to controls [8,9]. Yet another study showed AI acquisition assistance was also beneficial for experienced users obtaining cardiac POCUS windows [10]. However, fewer studies have evaluated the helpfulness of AI acquisition features for the Focused Assessment with Sonography for Trauma (FAST) exam, and there are inconsistent findings in the results of these prior studies [11,12].

In a randomized-controlled study of 30 non-physician medical providers, including nurses, nurse practitioners, and emergency medicine (EM) technicians without prior POCUS experience, participants who were randomly assigned to the intervention group utilized AI auto-grading for real-time feedback on image quality while obtaining the RUQ FAST exam window [11]. Each participant performed 10 exams on standardized patients. The median (interquartile range, IQR) American College of Emergency Physicians (ACEP) quality score was 5 (1) for the AI group and 4 (2) for the control group (p = 0.02) [13]. The study also reported a 14.36 (2.03 to 26.71) odds (95% confidence interval) that AI was associated with longer acquisition time. In a separate randomized-controlled study of 60 US Army medics without prior POCUS experience, participants who were randomly assigned to the intervention group utilized AI auto-labeling for the entire FAST exam [12]. No difference in median time (IQR) to acquire the FAST exam was reported with AI assistance (142 (87) vs 143 (84) seconds; p = 0.9). Average image quality (IQR) was higher on a 10-point scale in the AI group than the control group for the left-upper quadrant (9 (1.75) vs 8 (1); p = 0.01) and pelvic windows (10 (1.75) vs 8 (2.75); p < 0.01), but not the RUQ window (9 (2) vs 9 (3); p = 0.3).

These previous studies do provide some evidence to support the use of AI acquisition assistance features among novice users performing the FAST exam, though knowledge gaps remain. No study has evaluated the combined use of AI auto-labeling and auto-grading features among users obtaining FAST exam windows. Additionally, the impact of these features has not been studied in physician trainees of various experience levels to determine which users might receive the most benefit. Finally, no repeated-measures studies have been performed to investigate the immediate effect of these features within individual users obtaining FAST exam windows, as did our study investigating AI assistance in cardiac ultrasound acquisition [10]. A repeated-measures study evaluating the helpfulness of these features within individual users is important to guide use in isolated real-world events, such as performing a FAST exam on a trauma patient during a busy clinical shift.

Therefore, the objective of our repeated-measures study was to determine the immediate combined effect of AI auto-labeling and auto-grading on acquisition time and image quality of RUQ FAST exam windows obtained by both novice and experienced physician trainees. Our study focused specifically on the RUQ window, the most sensitive location for free fluid in the FAST exam, with the hope of clarifying the effect of AI assistance on RUQ acquisition, considering the inconsistent findings of prior studies [11,12,14].

## Materials and methods

Study participants

Fourteen novices, consisting of 12 first-year EM residents and 2 fourth-year medical students, and 10 experienced second- and third-year EM residents from a single program volunteered. The institutional review board (IRB) of Western Michigan University Homer Stryker M.D. School of Medicine (WMed) in Kalamazoo determined that the study met the criteria for an exemption.

Equipment

The Butterfly IQ+ probe (Butterfly Network, Inc., Burlington, MA, USA) was used with ScanLab software (Puchheim, Germany). AI acquisition assistance features included auto-labeling of abdominal organs and auto-grading of image quality.

Study protocol

The novice trainees received a 10-minute lecture and a 10-minute hands-on teaching session. The experienced participants had completed 2-3 years of a standard EM residency POCUS curriculum [13,15]. Participants acquired RUQ windows on three different standardized patients, both with and without AI assistance. The standardized patients included two female patients and one male patient between 45 and 55 years of age without known cardiac disease. The order of AI usage was randomly determined by the IRB staff in a permuted block fashion, and participants never examined the same patient twice in succession. The investigator adjusted the gain and depth, but no other assistance was provided. Participants notified the investigator when the best possible view had been achieved, and the investigator saved a six-second video clip.

Outcome measures

Acquisition time was defined as the total number of seconds the probe was contacting the standardized patient. Participants were unaware that the acquisition time was measured. Image quality was assessed by observing for three specific criteria: (i) essential structures visible (kidney, liver, Morrison’s Pouch, and the diaphragm), (ii) correct imaging plane (coronal plane with kidney in long axis without visualization of the inferior vena cava, gallbladder, or bowel), and (iii) proper probe orientation (indicator cephalad). The criteria were chosen to assess different image components for which the AI assistance might be beneficial.

Power analysis

An estimated sample size of 60 was needed to detect a 25-second difference in median acquisition time with a standard deviation of 30 at a 0.05 level of significance (alpha) and with 0.80 power (1-beta).

Data analysis

Acquisition times demonstrated rightward skew; therefore, median values and IQRs were determined, and Mann-Whitney U tests were performed. The investigators reviewing image quality were blinded to AI usage. One POCUS fellowship-trained EM physician and two senior resident EM physicians independently rated image quality. Each reviewer tabulated their observations as “yes” or “no.” Any disagreements were reviewed by the committee for accuracy. The final value for each observation used for statistical analysis was that which 2 of the 3 investigators initially indicated. No changes were made from this determination upon the committee review. Relative proportions with 95% confidence intervals (Wilson score) for the three image quality criteria with and without AI were determined. Pearson’s chi-square test and McNemar’s test for repeated-measures were used for comparison testing. All statistical tests were conducted using R Studio (Posit Software, Boston, MA, USA). The level of significance (alpha) for all statistical tests was 0.05.

## Results

A total of 143 RUQ windows were recorded. One recording from the novice users without AI assistance was missing and was excluded from the paired quality assessment. The median (IQR) acquisition time for novice trainees (69 (72) seconds) was longer than for experienced trainees (34 (30)), inclusive of all recordings (p < 0.01; Table 1). The median acquisition time was longer with AI assistance for both the novice (85 (91); 53 (59); p < 0.01) and the experienced trainees (44 (29); 28 (21); p = < 0.01). All RUQ quality criteria were significantly more likely among experienced than novice trainees (Table 2). No significant differences in image quality criteria with and without AI were observed within the novice or experienced trainee groups (Table 3).

**Table 1:** Acquisition time This table shows the median (IQR) time in seconds to acquire the right upper quadrant window with and without artificial intelligence, with p-values derived from the Mann-Whitney U test.

Study group	n	Median (IQR), seconds	p-value	U-statistic
Novice user recordings	83	69 (72)	<0.01	3,700
Experienced user recordings	60	34 (30)		
Novice users with AI	42	85 (91)	<0.01	1,100
Novice users without AI	41	53 (59)		
Experienced users with AI	30	44 (29)	<0.01	628
Experienced users without AI	30	28 (21)		

**Table 2:** Image quality by experience level This table shows the proportion (95% CI) of right upper quadrant recordings meeting quality criteria by experience level, including both AI and non-AI recordings, with p-values derived from Pearson’s chi-square test. df: degrees of freedom.

Criteria	Proportion (95% CI)		Chi-square test (df = 1)		
	Novice users	Experienced users	n	p-value	χ^2^
Essential structures	0.65 (0.54, 0.74)	0.92 (0.81, 0.97)	143	<0.01	14
Imaging plane	0.58 (0.47, 0.68)	0.78 (0.66, 0.87)		<0.01	6.6
Probe placement	0.78 (0.68, 0.86)	0.97 (0.88, 1.00)		<0.01	9.8

**Table 3:** Image quality with and without artificial intelligence assistance This table shows the proportion (95% CI) of right upper quadrant recordings obtained with and without artificial intelligence (AI) meeting quality criteria, with p-values derived from McNemar’s test for repeated measures. df: degrees of freedom.

Group	Criteria	Proportion (95% CI)		McNemar’s test (df = 1)		
		With AI	Without AI	n	p-value	χ^2^
Novice users	Essential structures	0.63 (0.48, 0.76)	0.67 (0.51, 0.79)	41	0.78	0.077
	Imaging plane	0.54 (0.39, 0.68)	0.62 (0.47, 0.75)		0.37	0.82
	Probe placement	0.83 (0.68, 0.92)	0.74 (0.59, 0.85)		0.25	1.3
Experienced Users	Essential structures	0.90 (0.73, 0.97)	0.93 (0.77, 0.99)	30	0.56	0.33
	Imaging plane	0.80 (0.62, 0.59)	0.77 (0.59, 0.88)		0.71	0.14
	Probe placement	0.97 (0.82, 1.00)	0.97 (0.82, 1.00)		1.00	0

## Discussion

This is the first repeated-measures study investigating the immediate combined effect of AI auto-labeling and auto-grading on acquisition time and image quality in both novice and experienced physician trainees obtaining the RUQ FAST exam window. We observed significantly longer acquisition times when AI features were used by both groups of trainees (Table 1). We theorize that the AI features encouraged trainees to spend more time attempting to obtain higher-quality windows. However, despite observing differences in image quality criteria by novice and experienced users, we found no differences in the image quality criteria within either group of trainees with or without AI (Table 3). We hypothesize that novice trainees did not have the probe manipulation skills to benefit from the AI auto-labeling and auto-grading. Conversely, the experienced trainees had minimal room for improvement with already high proportions of the quality criteria without AI assistance.

Two prior studies report inconsistent effects of AI assistance on RUQ acquisition time and image quality among novice non-physician trainees. One study by Chiu et al. reported longer RUQ acquisition time and improved image quality with auto-grading alone [11]. Another study by Hartline et al. reported no change in acquisition time for the entire FAST exam and no improvement in RUQ image quality with auto-labeling alone [13]. Uniquely, our study of physician trainees evaluated both auto-labeling and auto-grading together. Based on the collective results of the prior studies in conjunction with our results, we infer that auto-labeling alone may have a limited effect on acquisition time, but auto-grading is likely associated with longer acquisition time. Neither feature appears to be associated with a definitive improvement in image quality of the RUQ window.

There are notable differences between the two previous studies and our study that may contribute to the diverse results and must be considered when formulating conclusions about the usefulness of AI acquisition assistance for the FAST exam. Our study enrolled a group of physician trainees compared to the heterogeneous group of nurses, nurse practitioners, and EM technicians by Chiu et al. and the US Army medics by Hartline et al. [11,13]. Due to the varying training backgrounds, anatomy knowledge, and experience may have differed among the participants of the studies, which may have affected FAST exam performance and ultimately the helpfulness of AI assistance. However, the novice users in our study and the Army medics enrolled by Hartline et al. underwent a very similar FAST exam introductory session, comprised of a short lecture and hands-on practice session [12]. Lastly, the methods of image quality analysis differed in each study. We employed a binary “yes” or “no” system, Chui et al. utilized the ACEP five-point scale, and Hartline et al. created a 10-point scale [11]. Each system evaluated slightly different aspects of the ultrasound windows, limiting direct comparisons between the studies. 

The most distinctive feature of our study was the repeated-measures design, which was applied to both novice and experienced physician trainees. Our design provides further insight into the immediate effect of the AI acquisition assistance features when used in isolated attempts, such as performing a FAST exam in the midst of a clinical shift. Although our study was performed in a simulated setting, we hypothesize that AI acquisition assistance features would have limited benefit in isolated attempts to obtain RUQ FAST exam windows in clinical settings, but further study is needed.

Limitations

The small sample size is the most notable limitation of our study, and it increases the risk of type II error. Another limitation is the qualitative nature of our image quality analysis. A quantitative system, such as the ordinal ACEP grading scale used by Chiu et al. or the 10-point grading scale by Hartline et al., may have detected more subtle differences in image quality [11-13]. However, it is important to note that our grading system detected an expected difference in the quality of the RUQ recordings obtained by novice compared to experienced users, providing internal validity of our methodology. Finally, the repeated-measures design could have introduced learning bias as a confounding variable. Users may have remembered where to place the probe for the best quality image on their second examination of the same standardized patient. To limit learning bias, we randomized the order of AI usage.

## Conclusions

The immediate effect of AI auto-labeling and auto-grading was a longer acquisition time in both novice and experienced trainees obtaining RUQ FAST exam windows. AI acquisition assistance was not associated with an immediate improvement in image quality among novice or experienced trainees. Therefore, these AI features are unlikely to assist physician trainees with acquiring the RUQ FAST exam window in isolated attempts. Further study evaluating AI acquisition assistance for the other windows of the FAST exam and to validate these findings in a clinical setting is needed.
